# Influence of H_2_O_2_-Induced Oxidative Stress on In Vitro Growth and Moniliformin and Fumonisins Accumulation by *Fusarium proliferatum* and *Fusarium subglutinans*

**DOI:** 10.3390/toxins13090653

**Published:** 2021-09-15

**Authors:** Davide Ferrigo, Valentina Scarpino, Francesca Vanara, Roberto Causin, Alessandro Raiola, Massimo Blandino

**Affiliations:** 1 Department of Land, Environment, University of Padova, Agriculture and Forestry-AGRIPOLIS-Viale dell’Università 16, 35020 Legnaro, PD, Italy; davide.ferrigo@unipd.it (D.F.); roberto.causin@unipd.it (R.C.); 2 Department of Agricultural, Forest and Food Sciences, University of Torino, Largo Paolo Braccini 2, 10095 Grugliasco, TO, Italy; valentina.scarpino@unito.it (V.S.); francesca.vanara@unito.it (F.V.)

**Keywords:** *Fusarium* species, hydrogen peroxide, oxidative stress, mycotoxins, degradation

## Abstract

*Fusarium proliferatum* and *Fusarium subglutinans* are common pathogens of maize which are known to produce mycotoxins, including moniliformin (MON) and fumonisins (FBs). Fungal secondary metabolism and response to oxidative stress are interlaced, where hydrogen peroxide (H_2_O_2_) plays a pivotal role in the modulation of mycotoxin production. The objective of this study is to examine the effect of H_2_O_2_-induced oxidative stress on fungal growth, as well as MON and FBs production, in different isolates of these fungi. When these isolates were cultured in the presence of 1, 2, 5, and 10 mM H_2_O_2_, the fungal biomass of *F. subglutinans* isolates showed a strong sensitivity to increasing oxidative conditions (27–58% reduction), whereas *F. proliferatum* isolates were not affected or even slightly improved (45% increase). H_2_O_2_ treatment at the lower concentration of 1 mM caused an almost total disappearance of MON and a strong reduction of FBs content in the two fungal species and isolates tested. The catalase activity, surveyed due to its crucial role as an H_2_O_2_ scavenger, showed no significant changes at 1 mM H_2_O_2_ treatment, thus indicating a lack of correlation with MON and FB changes. H_2_O_2_ treatment was also able to reduce MON and FB content in certified maize material, and the same behavior was observed in the presence and absence of these fungi, highlighting a direct effect of H_2_O_2_ on the stability of these mycotoxins. Taken together, these data provide insights into the role of H_2_O_2_ which, when increased under stress conditions, could affect the vegetative response and mycotoxin production (and degradation) of these fungi.

## 1. Introduction

Moniliformin (MON) is an “emerging mycotoxin” with low molecular weight, mainly produced by several *Fusarium* species [1,2]. *Fusarium proliferatum* and *Fusarium subglutinans* are two plant pathogenic fungi known to produce a wide range of mycotoxins including MON [3], which is usually found in maize in association with fumonisins (FBs) [4,5]. MON is capable of causing disease in animals, interfering with mitochondrial respiration, progressive muscular weakness, respiratory distress, and cyanosis. Its acute toxicity is similar to that of T-2 and HT-2, the most toxic *Fusarium* mycotoxins [6,7,8]. Due to climate change and the temperature rise, the risk of mycotoxins produced by *Fusarium* spp. is expected to increase in the coming decades [9,10], involving the toxins produced by *Fusarium proliferatum* and *F. subglutinans*. At present, these two pathogens can be isolated under Southern European climate conditions, but the range of environments suitable for them to survive and colonize kernels is quite wide [11,12]. This characteristic, together with the aforementioned consequences of climatic changes, leads to the reasonable prevision of the spread of *F. proliferatum* and *F. subglutinans* toward Northern European regions, with a consequently increased risk of MON and FBs contaminations. Among the factors influencing mycotoxin production, stress factors are believed to have a great influence on mycotoxin biosynthesis [9,13,14], and a correlation is known to exist between environmental stress and mycotoxin biosynthesis [15,16,17]. In fact, in a variety of plants, mycotoxin production has been reported to be promoted by heat and drought stress [18,19], with a close connection between them, as there is some evidence that drought stress aggravation is just a consequence of the more conducive heat stress [18].

In response to stressors, both plants and fungi react with a rapid and transient release of reactive oxygen species (ROS), activating a broad range of strategies to protect themselves [20,21]. Among molecules acting as stress signals, hydrogen peroxide (H_2_O_2_) plays an important role; however, it is also involved in cell proliferation, differentiation processes [22], and the modulation of secondary metabolite production [23], such as mycotoxins. Previous experiments have shown that unfavorable growth conditions or culture supplementation with pro-oxidant compounds modulates mycotoxin production [24,25,26,27]. Indeed, in vitro increases of H_2_O_2_ concentration promoted aflatoxin production by *Aspergillus flavus* [28] and deoxynivalenol by *F. graminearum* while, to the contrary, nivalenol has been reported to decrease [29]. The increases of H_2_O_2_ concentration also influence FBs production by *F. verticillioides*, with an effect that is isolate-dependent [30]; however, to date, no data are available for MON. For these reasons, comprehension of the fungal response to ROS, such as H_2_O_2_, could be useful in developing effective strategies against mycotoxin contamination. The aim of this work was, therefore, to address whether oxidative stress mediated by H_2_O_2_ can modulate the survival and growth of *F. proliferatum* and *F. subglutinans*, as well as to evaluate its influence on the in vitro production of MON and FBs under mild stress conditions induced by hydrogen peroxide. Additionally, catalase activity, which has been found to be correlated with aflatoxin and trichothecene production by *A. flavus* and *F. graminearum* [29,31], was surveyed in these MON-producing isolates, due to its crucial role as an H_2_O_2_ scavenger. To simulate oxidative stress, treatments with H_2_O_2_ are commonly used [29,32,33,34,35]; however, its strong oxidizing properties could likely exert direct effects on molecules, such as mycotoxins. The use of oxidizing agents has been reported to act more or less effectively on several mycotoxins, performing a chemical transformation on them which can result in metabolites of lower or higher toxicity, depending on the agent and its oxidizing power [36]. Indeed, in the literature, data indicating the efficacy of ozone in degrading aflatoxin B_1_ [36,37], deoxynivalenol, FBs, ochratoxin, patulin, and zearalenone have been reported [36,38,39]. Although the detoxifying effect of H_2_O_2_ on MON has been reported at 5%, equivalent to ~1.47 M [40], no studies have assessed the low concentration of 1 mM. As H_2_O_2_ can play a concomitant and combined role on fungal growth, mycotoxin production, and structural modification of most mycotoxins, the role of H_2_O_2_ on MON and FBs degradation was also investigated.

## 2. Results

### 2.1. Effect of Temperature on Fusarium Growth

**Figure 1:**
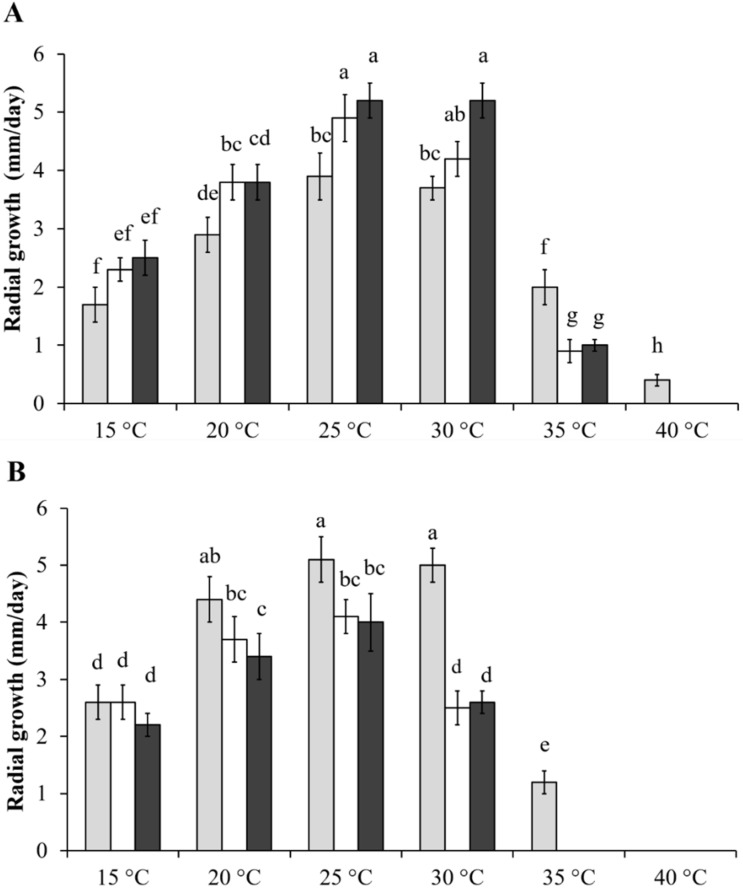
Daily radial growth of *F. proliferatum* and *F. subglutinans* isolates grown at different temperatures from 15 °C to 40 °C: (**A**) *F. proliferatum* isolates PRO1 (light grey bars), PRO2 (white bars), and PRO3 (dark grey bars); and (**B**) *F. subglutinans* isolates SUB1 (light grey bars), SUB2 (white bars), and SUB3 (dark grey bars). Values (mm ± SE; standard error) with different letters in columns are significantly different (*p* < 0.05), based on ANOVA and Tukey’s HSD tests. Statistical analyses were performed separately per species.

The estimation of fungal growth at different temperatures was performed by measuring the radial growth on PDA plates for both *Fusarium* species at temperatures ranging from 15–40 °C (Figure 1). The behavior of *F. proliferatum* at mild temperature was rather similar among isolates, with an optimal growth observed for each at 25–30 °C; however, when temperature increased to 40 °C, only the PRO1 isolate actively grew. With respect to the *F. subglutinans* isolates, the SUB1 isolate was able to grow at temperatures up to 35 °C, with an optimal range between 20 °C and 30 °C, while SUB2 and SUB3 were characterized by lower optimal temperatures of 20–25 °C and a maximum temperature of 30 °C. None of *F. subglutinans* isolates were able to grow at 40 °C. As fungal exposure to temperatures above the optimum range for growth may stimulate the rate of mitochondrial respiration and an increase in production of superoxide [41], the higher thermotolerance of *F. proliferatum*, with respect to *F. subglutinans* isolates, would also suggest a different behavior as a consequence of H_2_O_2_ oxidative treatment.

### 2.2. Effect of Hydrogen Peroxide Treatments on Fungal Biomass and Mycotoxin Content

The The effect of H_2_O_2_ supplementation on biomass of the *Fusarium* isolates was evaluated, and three main behaviors were observed with respect to the untreated control (see Table 1). The first behavior included isolates such as PRO1 and PRO2, having no or little effect with the increase of H_2_O_2_ concentration. The second one included isolates that were negatively influenced by oxidative stress increases, such as SUB1, SUB2, and SUB3, for whom the biomass was reduced by 47–58% at 10 mM. The last behavior included isolates that were positively influenced in biomass with an increase in H_2_O_2_; specifically, PRO3 obtained increases of 33% and 45% at 2 mM and 5 mM, respectively.

The effect of H_2_O_2_ was also investigated, in terms of its role in mycotoxin modulation (MON and FBs). To focus attention on a dosage which is able to induce a reasonable increase of intracellular H_2_O_2_ concentration at stress (>100 nM) [42] but not lethal levels [43], treatments were carried out at 1 mM H_2_O_2_. Mycotoxin quantification in tested isolates (Table 2) revealed a low production of MON by *F. proliferatum* isolates (0.018–7.843 ng/mg), while a low content of FBs was produced by *F. subglutinans* isolates (2.625–7.120 ng/mg). The effect of 1 mM H_2_O_2_ supplementation on MON and FB fungal production is reported in Table 2. The mycotoxin content was normalized to dry weight fungal biomass, and the effect of H_2_O_2_ (with respect to the untreated control) showed that the isolates PRO2, PRO3, and SUB1 were not significantly influenced by H_2_O_2_ treatment, in terms of the MON content. In a similar way, PRO2, PRO3, SUB1, SUB2, and SUB3 were not significantly affected by the effect of H_2_O_2_, in terms of FBs content; although, in most cases (PRO2, SUB1, SUB2, SUB3), a reduction—ranging from 60 to 98% FB content—was been recorded, and only for PRO3 was an increased concentration (+15%) of FBs observed. Considering the MON production in PRO3-, SUB2-, and SUB3-inoculated media, it was significantly reduced (more than 97%) after treatment with 1 mM H_2_O_2_. FBs production of treated PRO1 was likewise significantly reduced (by 64%).

### 2.3. Effect of Hydrogen Peroxide Treatments on Catalase Activity

**Figure 2:**
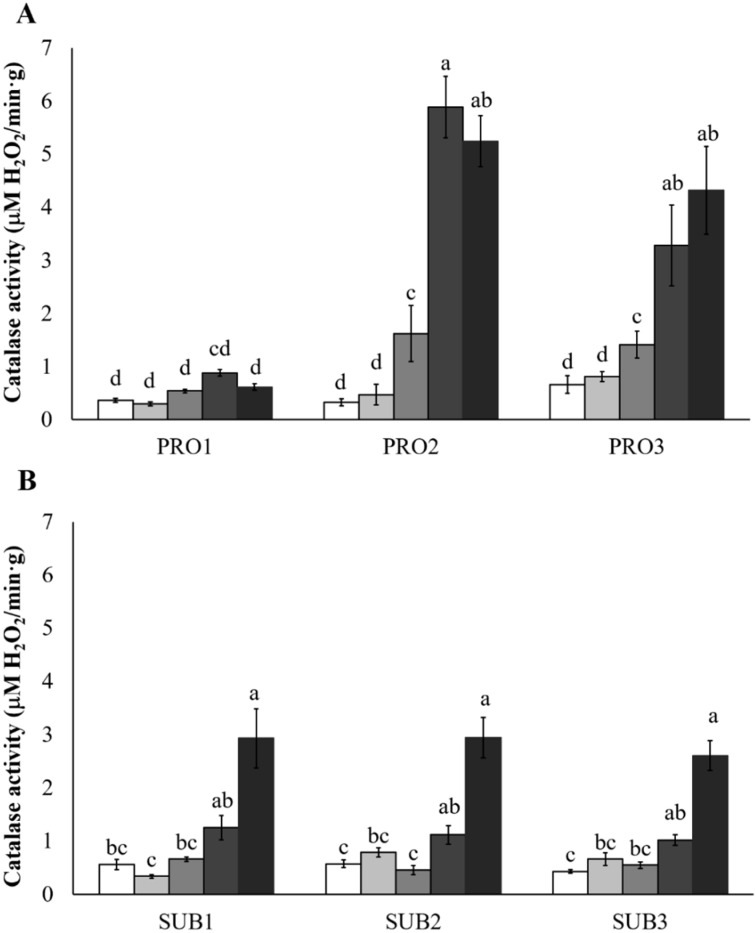
Catalase activity of *F. proliferatum* ((**A**); PRO1, PRO2, and PRO3) and *F. subglutinans* ((**B**); SUB1, SUB2, and SUB3) isolates at different H_2_O_2_ concentrations after H_2_O_2_ treatment (untreated control, white bar; 1 mM, light gray bar; 2 mM, grey bar; 5 mM, dark grey bar; and 10 mM, black bar). Activity is expressed as micromoles of H_2_O_2_ consumed per minute per gram of dry fungal biomass. Bars ± SE (Standard Error) with different letters are significantly different (*p* < 0.05), based on ANOVA and Tukey’s HSD tests.

Note that activity is expressed as micromoles of H_2_O_2_ consumed per minute per gram of dry fungal biomass. Catalase activity, surveyed after treatment, revealed different behaviors between species and isolates (Figure 2). With respect to *F. proliferatum* isolates, the activity for PRO2 and PRO3 differed from their respective control culture, starting from 2 mM, while a quick increase appeared for 5 mM and 10 mM H_2_O_2_ treatments. Maximum values of 5.89 and 4.32 were observed for PRO2 and PRO3 treated with 5 and 10 mM H_2_O_2_, respectively. On the other hand, PRO1 was observed to not be influenced by any H_2_O_2_ concentration, with a maximum activity of 0.88. Concerning *F. subglutinans*, catalase activity increased with low slope as the H_2_O_2_ concentration increased; thus, no differences were observed among the untreated control, 1, and 2 mM H_2_O_2_ treatments. The same trend was observed after 5 and 10 mM H_2_O_2_ treatments. Moreover, no difference was observed among *F. subglutinans* isolates at each evaluated concentration, with the highest values detected at 10 mM (2.60–2.93).

### 2.4. Effect of 1 mM Hydrogen Peroxide on MON and FBs in a Certified Maize Reference Material and in a Multi-Mycotoxin Analytical Standard Solution

The effect of H_2_O_2_ (1 mM) in a certified maize reference material and in a multi-mycotoxin analytical standard solution of MON and FBs, with respect to an untreated control, was evaluated in order to validate the experimental plan and to understand the impact of H_2_O_2_, as an oxidizing agent, on the chemical structure of MON and FBs. For this purpose, we tested the stability of MON and FB both in a neat solvent (standard solution) and in the maize matrix, preparing the multi-mycotoxin standard solution at the same concentration value for both MON and FBs. The results are reported in Table 3. MON and FBs were significantly reduced in both of the matrices, neat solvent, and maize matrix. Indeed, MON was completely reduced (−100%) in the standard solution and strongly reduced (−71%) in the certified maize reference material. Similarly, but with a lower efficacy, FBs were reduced by 56% in the standard solution and by 76% in the maize matrix.

## 3. Discussion and Conclusions

Reactive oxygen species play a pivotal role in regulation of primary and secondary metabolism in fungi [14,23], acting as stress signal, modulating antioxidant activity during plant–fungi interactions [44,45,46], and facilitating mycotoxin production [47,48]. Among the emerging mycotoxins, the health concerns for MON produced by *F. proliferatum* and *F. subglutinans* has been ascribed increasing importance. Due to their different environmental needs [12,49,50], *F. proliferatum* is more common in warm areas of southern Europe, including Italy [5]. However, the spread of *F. subglutinans* from the humid and cool areas of north-western and central Italy [3,51] could represent a potential risk for MON contamination in the future, as a consequence of climate change. A direct result of heat stress is the production of ROS and the induction of antioxidant defense [52], where ROS scavengers such as catalase can serve as determinants for fungal tolerance to such stress [53]. Preliminary information on growth response to temperature highlighted the variable behavior among mycotoxigenic species and isolates. *F. proliferatum* isolates have demonstrated a large variability in fungal growth under different environmental parameters, such as temperature [54], while specific data regarding *F. subglutinans* are limited [49,55]. The data presented in this paper indicate that the investigated isolates were able to grow at higher temperatures, compared to those where they were isolated (June–August, 28.5°). Concerning *F. proliferatum*, the radial growth of the isolate PRO1 up to 30 °C was lower, when compared to PRO2 and PRO3, but PRO1 showed the best values at 35 °C. Previous studies conducted on *Zymoseptoria tritici* have reported that strains that grew faster under a favorable environment were the most sensitive to oxidative stress [56]. Our data—at least, those for *F. proliferatum*—confirmed these findings as PRO1, with the lowest growth rate in the range 15–30 °C, was the fastest to grow at 35° and the only one able to survive at 40 °C. Concerning the three *F. subglutinans* isolates, their growth was optimal in the range 20–25 °C, with an average decrease of 25% at 30 °C. The best performance among *F. subglutinans* was observed for the SUB1 isolate, the which was the only isolate able to survive at 35 °C. The presented data, therefore, strengthen previous findings on the warmer environmental conditions required by *F. proliferatum* than *F. subglutinans* [12], but also highlight the presence of isolates characterized by adaptability to high temperature (and, thus, oxidative stress), and the possibility of a selective pressure to new conditions, as has been observed for other pathogens [57,58]. Fungal growth can be influenced in a direct manner due to the activity of oxidants on cell components and, in an indirect way, as a complex of physiological responses to oxidative stress. Among the direct effects on fungal tissues, high molecular weight polysaccharides (i.e., α- and β-linked glucans and chitin), which represent the main structure of filamentous fungi cell walls, are not directly affected by H_2_O_2_, although this oxidant species is required for the degradative activity of some enzymes [59,60]. On the other hand, H_2_O_2_ is responsible for the reversible or irreversible oxidation of wall-bound glycoproteins and, moving across the cell membranes, can damage inner lipids and peptides, altering their biological function and leading to cell death [61] and biomass reduction.

Comparison of the data obtained regarding fungal survival and biomass growth after H_2_O_2_ treatment revealed no apparent relations. Generally, as has been observed in other studies, when oxidative stress increased, fungal growth was reduced [30,32,62]. The isolates PRO1 and PRO2 did not decrease their biomass with increasing H_2_O_2_ treatments, while PRO3 growth was promoted, suggesting tolerance to high oxidative conditions. Similar considerations can be drawn for the *F. subglutinans* isolates; in fact, the negative impact of H_2_O_2_ on the temperature-tolerant SUB1 was not different to that of the more temperature-sensitive SUB2; to the contrary, SUB3 was negatively influenced by high temperature, but not negatively influenced by H_2_O_2_ (except for the 10 mM H_2_O_2_ treatment). The biomass increase observed for some isolates, as a consequence of a low oxidative treatments, has already been observed for *Sclerotium roolfsi* and *Sclerotinia sclerotiorum* where H_2_O_2_, supplied at the concentration of 1 mM, was associated to a mass of highly proliferating interwoven hyphae [42]. Indeed, it has been suggested [63] that one of the possible outcomes of a hyperoxidant state could be an increased reducing power derived from nutrient utilization to compensate for ROS levels. For these reasons, it is possible to suppose that, as a side-effect of the activity of H_2_O_2_ on tissues, the increased biomass could be the fungal attempt to balance the exogenous oxidative stress by consuming nutrients that provide reducing power. The fact that this aspect occurred for some isolates could be dependent on the specific thermotolerance of the strain, determined by its inherent growth capability and genetic traits (QTL) that make these isolates better- or worse-performing under stress conditions [56]. Fungi possess more or less efficient oxidant scavenging capabilities, depending on the species [35,42], isolate, and chemotype [29,56], or on physiological adaptation [64]. The above-mentioned variables could, therefore, explain the different effects that an identical H_2_O_2_ concentration produced in the surveyed pathogens. Among stress-scavenging systems, catalases are crucial enzymes in the cellular defense against H_2_O_2_ [65,66], and their activity could provide further insights regarding the influence of oxidative stress on *F. proliferatum* and *F. subglutinans*. Although catalase activity increased with an increase in temperature in an *Aspergillus niger* strain [52], the different tolerances to temperature and H_2_O_2_ concentrations by *Fusarium* spp. tested in this study were not clearly correlated with differing trends in catalase activity. In fact, catalase activity in *F. subglutinans* did not differ among isolates with different thermotolerance. On the other hand, with exception of PRO1, the ready and stronger catalase response by *F. proliferatum* isolates can suggest their better adaptation to oxidative stress, compared to the *F. subglutinans* isolates. This fact could be supported by the more frequent environmental stress that *F. proliferatum* can encounter, such as hot temperatures and drought conditions, with respect to *F. subglutinans*. Moreover, in the present study, catalase activity was enhanced starting from 2 mM for *F. proliferatum* and 5 mM for *F. subglutinans* and, although the behavior of these isolates were expected to confirm oxidative tolerance with respect to the other *Fusarium* spp., comparable studies with five *F. graminearum* and *F. culmorum* strains [29] have shown that 0.5 mM H_2_O_2_ treatment was sufficient to modify catalase activity. Therefore, comparison with a species with similar environmental requirements such as *F. subglutinans* remains unmatched.

Concerning the production of the two toxins in growth media, the data indicated that isolates with high toxigenic potential (i.e., SUB2 and SUB3 for MON; PRO1 for FBs) significantly reduced (~99% for MON; ~64% for FBs) the mycotoxin content after treatment with 1 mM H_2_O_2_. In all other cases, the H_2_O_2_ treatment resulted in a non-significant effect; on the other hand, interestingly, SUB1 for MON and PRO3 for FBs seemed not to be negatively influenced by the treatments and the content of toxins in the treated samples was higher than in the untreated control, but not statistically different. However, mycotoxin content analyzed in a certified maize reference material and in artificially contaminated neat solvent reported similar results, both in the presence and absence of pathogen activity. These results suggest an effect on the surveyed mycotoxin which is not mediated by fungal metabolism, thus revealing a possible direct degradation or modification of MON and FBs molecules by H_2_O_2_. As previously discussed, the addition of H_2_O_2_ to simulate a stressful environment also produced a degradative effect towards MON and FUM production that, therefore, cannot be attributable to the sole biosynthesis modulation induced by stress. For the same reasons, as MON and FBs content of SUB1 and PRO3 did not decrease, as occurred for the other samples, it is possible to hypothesize that the degradative effect by treatment could be compensated for by the promotion of mycotoxin biosynthesis. An influence of oxidative stress on the biosynthesis of MON and FUM in *F. proliferatum* and *F. subglutinans* cannot be excluded, as has been observed for other toxigenic fungi [62,67]. The differential behaviors towards oxidative stress and the biosynthesis of mycotoxins observed in present work suggest that, as has been previously observed in *F. verticillioides* isolates [30], the effect of H_2_O_2_ treatment on mycotoxin production could be strain-dependent. The involvement of a direct effect could be further supported by fungal catalase activity which, without significant changes after 1 mM H_2_O_2_ treatment, is unlikely to be associated to MON and FB changes, as has been suggested for other fungi [68]. However, we cannot exclude that catalases may play a role in the modulation of mycotoxin production under high-stress conditions, as represented by the exposure at 5 and 10 mM H_2_O_2_—levels which are difficult to reach in a sole stressful environmental condition. It could be assumed that the observed decrease of MON content could be linked to disappearance of the double bond and an opening of the 4-C ring structure. Similarly, in the same matrices, FBs showed a decrease in their content, probably due to an oxidizing transformation with the formation of 3-keto derivatives of FBs [69].

Taken together, these data highlight that the effect of oxidative stress induced by H_2_O_2_ in mycotoxigenic *Fusarium* species cannot be evaluated simply in terms of the modulation of toxin biosynthesis, but more likely as a dynamic interaction between the fungal isolate, indirect mycotoxin response to oxidative stress, and direct mycotoxin degradation or modification. For these reasons, no clear explanations may be inferred about the effect of H_2_O_2_ on the modulation of MON and FBs biosynthesis in *F. proliferatum* and *F. subglutinans*, while the low stability of these toxins upon treatment more likely appeared. It could be interesting, in the future, to assess the kinetics of mycotoxin (MON and FBs) production by the different fungal isolates and the kinetics of degradation or modification of the mycotoxins produced, in order to understand which is the dominant effect between the stimulation of mycotoxin production or the degradative effect of H_2_O_2_ on the produced mycotoxins, which is eventually not completely metabolized by the isolates and remains present in the culture medium. The complexity of factors involved in this interaction suggests that the influence of stressors on mycotoxin modulation should be better investigated through the use of pro-oxidant compounds, such as menadione, paraquat, or fungicide molecules [70,71,72,73,74], which should not interact directly (even at low concentration) with the mycotoxin content. To our knowledge, this is the first time that an effect of H_2_O_2_ on MON production by two of its main fungal producers has been assessed. To date, no data have been reported regarding the use of H_2_O_2_ as a chemical agent to detoxify or decontaminate MON at the concentration used in this study, and the few studies focused on FBs detoxification or degradation are mainly due to the use of oxidizing agents such as ozone [36,39,69,75], while more data are available for the detoxification of aflatoxin [76], ochratoxin A [77], and zearalenone [78]. However, it is important to highlight that the degradation of mycotoxins did not always correlate with detoxification but, rather, it depends on the toxicity of the derived chemical products. In fact, although chemical treatments have been shown to be effective in the reduction of mycotoxin content, they could cause some irreversible changes and leave residues on the food/feed, or convert the structure of the mycotoxin into another compound with unknown structure and toxicity [79].

In conclusion, the data obtained from this study provide further insight into the adaptability of *F. proliferatum* and *F. subglutinans* to increasing oxidative stress mediated by high temperature, and that their behavior partially correlates with fungal growth and increased catalase activity under these conditions. Unfortunately, no definitive conclusions can be drawn, with respect to the biological effect of H_2_O_2_ on mycotoxin production. On the other hand, the findings of the present work improve the understanding of the role of the exogenous application of H_2_O_2_ when studying mycotoxins under simulated oxidative stress conditions, considering the potential direct side-effect of this molecule, which should be taken into account. Further insights need to be derived, with the help of advanced analytical techniques and toxicity studies, on the chemical degradation products that could result due to fragmentation or modification of MON and FBs after H_2_O_2_ treatment, in order to elucidate their molecular structures and level of toxicity.

## 4. Materials and Methods

### 4.1. Chemicals

Methanol (CH_3_OH), acetonitrile (CH_3_CN), and water (H_2_O) were LC gradient grade or LC-MS grade, depending on their use during the extraction or analytical phases, and were purchased from VWR (Milan, Italy). Glacial acetic acid (CH_3_COOH) was obtained from Sigma-Aldrich (St. Louis, MO, USA). Stock solutions of MON and fumonisins (FBs, FB_1_ and FB_2_) were respectively prepared in CH_3_CN/H_2_O (90/10 *v*/*v*) and in CH_3_CN/H_2_O (50/50 *v*/*v*) (Romer Labs Diagnostic GmbH, Tulln, Austria). Two composite standard working solutions were prepared by dissolving appropriate volumes of each analyte in a dilution phase mixture, CH_3_CN/H_2_O (50/50, *v*/*v*), as described by Scarpino [80]. These two working solutions were then mixed in appropriate volumes, in order to prepare the working solutions for calibration. All the solutions were stored at −20 °C in amber glass vials, and were brought to room temperature before use.

### 4.2. Fusarium Isolates Identification and Inoculum Preparation

Three *F. proliferatum* (named PRO1, PRO2, and PRO3) and three *F.* *subglutinans* (named SUB1, SUB2, and SUB3) colonies were isolated from maize kernels grown in Carmagnola (Piedmont, Italy) and morphologically characterized according to Leslie [81]. Single-spore cultures were obtained for each isolate [82] and, in order to confirm the identification at the species level, *Fusarium* isolates were submitted to PCR with primer pairs designed by Mulè et al. [83] on the calmodulin gene for *F. proliferatum* (PRO1/2; ~585 bp product) and *F. subglutinans* (SUB1/2; ~631 bp). Similar species (i.e., *F. temperatum* or *F. langsethiae*) were properly excluded by means of PCR analysis with apposite primers (data not shown). Conidial suspensions were obtained by growing fungal cultures on SNA (Spezieller Nahrstoffarmer Agar) at 25 °C under dark conditions for 8 days before spore collection with sterile water. Conidial suspensions were quantified and diluted to obtain a final concentration of 10^8^ conidia per milliliter. All chemicals for medium preparation were obtained from Sigma-Aldrich (St. Louis, MO, USA).

### 4.3. Effect of Temperature on Fusarium Growth

Environmental conditions at the isolation site (Carmagnola, Piedmont, Italy) were registered from June to August during 2015–2019, reporting an average maximum temperature of 28.5 °C (June 26.9 ± 1.2°C; July 29.8 ± 1.2; August 28.8 ± 0.7 °C). In this context, a thermal condition of particular interest seems to be 30 °C, as it represents the optimal temperature for MON production in *F. subglutinans* [55], and a 1.5–2 °C rise in temperature represents the most probable scenario of climate change expected for the coming years in Southern Europe [84]. The tolerance of the tested pathogens to rising temperatures was assessed. Petri plates containing potato dextrose agar (Difco PDA, Sparks, MD, USA) were inoculated in the center with a plug of fresh mycelia each (5 mm in diameter) and grown in the dark at 15, 20, 25, 30, 35, and 40 °C. Radial growth was assessed daily for a maximum of five days. Ten plates were replicated for each temperature. Radial growth data are presented as millimeters per day.

### 4.4. Effect of Hydrogen Peroxide Treatments on Fusarium Biomass and Mycotoxin Production

For each isolate, 100 μL of a conidial suspension (10^8^ conidia/mL) was inoculated in 100 mL of maize meal broth, in order to provide the same carbon sources and nutrients as in the field. Maize meal broth was prepared according to Nakamoto [85], with some modifications. Briefly, maize grains (KERIDOS dent hybrid, KWS Italia, FAO 600) with no detectable mycotoxins were milled and homogenized with a Grindomix GM200 (Retsch, 10,000 rpm, 30 s pulse for two times). Ten grams of maize meal per liter were mixed with cold distilled water and continuously stirred overnight at room temperature. The next day, insoluble components were removed by filtration, the filtrate was autoclaved and its pH was adjusted to 6.5. Inoculated cultures were grown in the dark at 30 °C on an oscillatory shaker (150 rpm) for 3 days before H_2_O_2_ treatment. This specific temperature was well-tolerated by all the isolates, and it has been reported as optimal for MON production in *F. subglutinans* [55]. Treatments consisted of H_2_O_2_ (30% *w*/*w*; Sigma-Aldrich, St. Louis, MO, USA) supplementation every 24 h, to obtain a final concentration of 1, 2, 5, or 10 mM. Non-treated cultures were supplemented with sterile water and used as control. Fungal cultures were grown according to above-mentioned conditions for a total of 21 days. Fungal biomass was separated by supernatant, thoroughly washed with tap water and excess water was squeezed out. Biomasses were oven-dried (100 °C for approximately 48 h) and weighed to analyze the effect of H_2_O_2_ treatment on fungal growth. To evaluate the mycotoxin production, fungal cultures were grown as described above and treated daily with 1 mM H_2_O_2_. After incubation, the supernatant was filtered through Whatman paper No. 4 and 50 mL were added to 50 g of toxin-determined maize flour and mixed. Flour samples were dried overnight at 40 °C and homogenized before LC-MS/MS multi-mycotoxin analysis. Fungal weight was used to normalize the toxin content with respect to biomass. Each experiment was performed at least in three replicates.

### 4.5. Effect of Hydrogen Peroxide Treatments on Fusarium Catalase Activity

Fungal cultures were grown as described above and treated daily with H_2_O_2_ for 5 days before biomass collection. Each experiment was performed in at least three replicates. Fungal biomass was thoroughly washed with tap water and vacuum separated to remove the liquid broth. For each culture, 100 mg biomass was suspended in 1 mL of buffer (phosphate buffer 50 mM, EDTA 1 mM; pH 7.0) and maintained in an ice-bath to be disrupted by sonication. Sonication was set up with some modifications, according to Klimek-Ochab [86] and performed using a Sonic Dismembrator FB50 (Fisher Scientific Co., Pittsburgh, PA, USA). Cell disruption was obtained by the application of acoustic waves (40% power; 8 cycles composed of 15 s sonication, 15 s ice cooling). The homogenate was then centrifuged (Rotina 35R, 10,000 rpm,10 min, 4 °C; Hettich Zentrifugen) and the cell extract was collected for analysis. Catalase activity was determined using the method of Hadwan and Abed [87] with some modifications. The reaction mixture contained 5 mM H_2_O_2_ in 50 mM phosphate buffer at pH 7.0 and 200 μL of free-cell extract. The reaction mixture was incubated for 4 min at 25 °C and stopped with ammonium molybdate (32.4 mM). Decomposition of H_2_O_2_ was followed by measurement of absorbance at 374 nm of the yellow complex of molybdate and quantified using an external calibration curve obtained with H_2_O_2_ standard solutions. Activity is expressed as micromoles of H_2_O_2_ consumed per minute per gram of dry fungal biomass. Each biological sample was submitted to catalase determination in three technical replicates.

### 4.6. Effect of Hydrogen Peroxide on MON and FBs in a Certified Maize Reference Material and in a Multi-Mycotoxin Analytical Standard Solution

The effect of H_2_O_2_ on MON and FBs was assessed in two matrices:

- in a certified reference material of maize containing certified concentrations of FBs (FBs = FB_1_ + FB_2_ = 2600 ± 278 µg/kg) and measured concentration of MON (Trilogy^®^ Analytical Laboratory, Washington, MO, USA)
- in a multi-mycotoxin analytical standard solution containing a comparable concentration of FBs and MON to that of the certified reference material of maize.

Each of the two matrices were treated with 1 mM H_2_O_2_ in the following way:

- Five grams of the certified reference material of maize were treated with 5 mL of H_2_O_2_ in a conical flask and incubated for 72h at 30 °C. After this incubation period the weight was checked and the sample was extracted with 15 mL of extracting solution CH_3_CN/H_2_O/CH_3_COOH (79/20/1, *v*/*v*/*v*) and subsequently underwent to the other extraction and dilution steps.
- the multi-mycotoxin analytical standard solution with a comparable concentration of FBs and MON to that of the certified reference material of maize was prepared in CH_3_CN/H_2_O, 50/50 (*v*/*v*) containing 1 mM H_2_O_2_, not acidified with CH_3_COOH, in order to preserve the oxidizing capacity of H_2_O_2_, which is essential to assess the effect of H_2_O_2_ as a presumable degrading agent on MON and FBs.

The two matrices treated with 1 mM H_2_O_2_ were compared to an untreated control. Each experiment was performed in three replicates and all the samples have been subjected to LC-MS/MS multi-mycotoxin analysis for the determination of MON and FB content.

### 4.7. LC-MS/MS Multi-Mycotoxin Analysis for the Determination of MON and FB Content

The extraction and sample preparation was performed by applying the dilute-and-shoot method reported by Scarpino [80]. Briefly, 5 g of flour samples obtained after the inoculum of *Fusarium* biomass treated or not treated with 1 mM H_2_O_2_ was extracted by mechanical shaking at 300 rpm for 90 min (shaker mod. RS-LS 20, Phoenix Instrument, Garbsen, Germany) with 20 mL of extracting solution CH_3_CN/H_2_O/CH_3_COOH (79/20/1, *v*/*v*/*v*). The extract was filtered through Whatman^®^ grade 1 filters (Brentford, UK) and subjected to dilution with the same volume of diluting solution (CH_3_CN/H_2_O/CH_3_COOH 20/79/1, *v*/*v*/*v*). The diluted extract was vortexed and filtered through 15 mm diameter, 0.2 µm regenerated cellulose (RC) syringe filters (Phenex-RC, Phenomenex, Torrance, CA, USA). After appropriate mixing, 20 µL of the diluted filtered extract was analyzed without any further pre-treatment.

LC-MS/MS analysis was carried out on a Varian 310 triple quadrupole (TQ) mass spectrometer (Varian, Italy), equipped with an electrospray ionization (ESI) source, a 212 LC pump, a ProStar 410 AutoSampler, and dedicated software. Liquid chromatography (LC) separation was performed on a Gemini-NX C18 100 × 2.0 mm i.d., 3 μm particle size, 110 Å, equipped with a C18 4 × 2 mm security guard cartridge column (Phenomenex, Torrance, CA, USA). The mobile phase consisted of two eluents: water (eluent A) and methanol (eluent B), both of which were acidified with 0.1% *v*/*v* CH_3_COOH delivered at 200 µL/min. In order to quantify all the analytes with positive and negative polarity, two separate chromatographic runs per sample were carried out. The chromatographic conditions of the runs for the negative and positive ionization mode acquisitions have been previously described in detail [80].

Mass spectrometric analysis (ESI-MS/MS) was performed in selected reaction monitoring (SRM) mode, alternating two transition reactions for each compound in both negative and positive ionization modes, in two separate chromatographic runs per sample. The mass spectrometric parameters have been more thoroughly described in [80].

Results pertaining to the linearity range, the limit of detection (LOD), the limit of quantification (LOQ), the apparent recovery (R_A_, %), the matrix effects through the evaluation of the signal suppression/enhancement (SSE, %), and the recovery of the extraction (R_E_, %) have been previously reported for all of the analyzed mycotoxins [80].

### 4.8. Statistical Analysis

Values of fungal growth (radial growth and biomass) were log-transformed before statistical analysis, while the mycotoxin content from the pathogenic and mycotoxigenic trial was normalized on the basis of dry weight fungal biomass. Percent variance values were calculated on the basis of the mycotoxin concentration variation (%), comparing the value after treatment with 1 mM H_2_O_2_ with the value of the control for each isolate and mycotoxin for both trials (4.5 and 4.6). Statistical analysis of the effect of H_2_O_2_ supplementation on *Fusarium* isolate growth, catalase activity, and mycotoxin content were performed by ANOVA, followed by Tukey’s HSD test. These results were computed from experiments performed in triplicate. Data on dry biomass and mycotoxin content were expressed as percent values relative to the untreated control. Analyses were conducted using the XlStat 2016 software.

## Figures and Tables

**Table 1:** Fungal Biomass Content of *Fusarium proliferatum* (PRO) and F. Scheme 2. O2.

	PRO1		PRO2		PRO3	
H_2_O_2_ Concentration	Biomass (mg ± SE)	Relative Yield%	Biomass (mg ± SE)	Relative Yield%	Biomass (mg ± SE)	Relative Yield%
Control	1298 ± 153 ^a^	100	860 ± 80 ^a^	100	1701 ± 88 ^bc^	100
1 mM	1275 ± 74 ^a^	98	809 ± 47 ^a^	94	1616 ± 55 ^c^	95
2 mM	1362 ± 27 ^a^	105	832 ± 63 ^a^	97	2265 ± 107 ^a^	133
5 mM	1246 ± 52 ^a^	96	920 ± 79 ^a^	107	2460 ± 312 ^a^	145
10 mM	1220 ± 132 ^a^	94	958 ± 64 ^a^	111	1841 ± 181 ^ab^	108
	**SUB1**		**SUB2**		**SUB3**	
**H_2_O_2_** **Concentration**	**Biomass (mg ± SE)**	**Relative Yield %**	**Biomass (mg ± SE)**	**Relative Yield%**	**Biomass (mg ± SE)**	**Relative Yield%**
Control	528 ± 27 ^a^	100	1047 ± 16 ^a^	100	1008 ± 71 ^ab^	100
1 mM	385 ± 53 ^b^	73	635 ± 73 ^ab^	60	970 ± 73 ^ab^	96
2 mM	417 ± 89 ^ab^	79	693 ± 22 ^ab^	66	1210 ± 112 ^a^	120
5 mM	370 ± 52 ^bc^	70	534 ± 43 ^b^	51	1330 ± 96 ^a^	132
10 mM	280 ± 33 ^c^	53	440 ± 81 ^b^	42	443 ± 39 ^c^	44

Dry weight fungal biomass expressed as percent values relative to their level in the control culture. Values ± SE (Standard Error) with different letters on the columns are significantly different (*p* < 0.05) based on ANOVA and Tukey′s HSD tests. Statistical analyses were performed separately per isolate.

**Table 2:** Moniliformin (MON) and fumonisin (FBs = FB1 + FB2) content after treatment of conidial suspension of *Fusarium proliferatum* (PRO) and *F. subglutinans* (SUB) isolates with H_2_O_2_ (1 mM).

Isolate	MON ^a^				FBs ^a^			
	ng/mg ± SE		*p*-Value ^b^	Percent Variance (%) ^c^	ng/mg ± SE		*p*-Value ^b^	Percent Variance (%) ^c^
	Control	H_2_O_2_			Control	H_2_O_2_		
PRO1	7.843 ± 0.971	0.010 ± 0.002	**	−99.9	937.584 ± 34.583	337.061 ± 30.139	**	−64.1
PRO2	0.018 ± 0.002	0.019 ± 0.001	ns	5.1	288.672 ± 118.101	112.375 ± 36.661	ns	−61.1
PRO3	0.299 ± 0.290	0,009 ± 0.001	ns	−96.9	448.426 ± 45.825	517.013 ± 24.130	ns	15.3
SUB1	0.029 ± 0.002	0.040 ± 0.005	ns	41.2	7.120 ± 6.195	0.282 ± 0.034	ns	−96.0
SUB2	49.654 ± 9.950	0.097 ± 0.073	**	−99.8	2.625 ± 2.525	0.170 ± 0.020	ns	−93.5
SUB3	144.156 ± 41.304	3.681 ± 3.665	*	−97.4	6.128 ± 3.021	0.108 ± 0.001	ns	−98.2

Statistical analyses were performed separately per isolate and per mycotoxin. Reported data were the average of 3 replications and were expressed in ng/mg ± SE (Standard Error). ^a^ The mycotoxin content means were normalized on dry weight fungal biomass. ^b^ *p*-value = level of significance of ANOVA, ns = *p* > 0.05, * = *p* < 0.05, ** = *p* < 0.01. ^c^ Percent variance values were calculated on the basis of the mycotoxin concentration variation (%) comparing the value after treatment with H_2_O_2_ 1 mM with the value of the control for each isolate and mycotoxin.

**Table 3:** Effect of treatment with H_2_O_2_ (1 mM) on MON and FBs (sum of FB_1_ and FB_2_) in a certified maize reference material (Certified concentration for FBs = 2.600 ± 0.278 ng/mg, Trilogy^®^ Reference Material, Trilogy^®^ Analytical Laboratory, Washington, MO, USA) and in a multi-mycotoxin analytical standard solution prepared at the same concentration value for both MON and FBs.

Sample	MON				FBs			
	ng/mg ± SE		*p*-Value ^a^	Percent Variance (%) ^b^	ng/mg ± SE		*p*-Value ^a^	Percent Variance (%) ^b^
	Control	H_2_O_2_			Control	H_2_O_2_		
Multi-mycotoxin standard	1.763 ± 0.069	<LOD ^c^	***	−100.0	2.639 ± 0.054	1.164 ± 0.025	***	−55.9
Certified maize Reference Material	1.755 ± 0.021	0.505 ± 0.014	***	−71.2	2.720 ± 0.088	0.645 ± 0.051	***	−76.3

Reported data were the average of 3 replications and were expressed in ng/mg ± SE (Standard Error). ^a^ *p*-value = level of significance of ANOVA, *** = *p* < 0.001. ^b^ Percent variance values between the mycotoxin concentration after the treatment with H_2_O_2_ and the concentration value in the control. ^c^ LOD MON = 0.0003 ng/mg.

## Data Availability

The authors declare that the data supporting the findings of this study are available within the paper. Raw data are available from the corresponding author upon reasonable request.

## Abbreviations

moniliformin (MON), fumonisins (FBs), hydrogen peroxide (H_2_O_2_), reactive oxygen species (ROS), LC-MS/MS (liquid chromatography-mass spectrometry), methanol (CH_3_OH), acetonitrile (CH_3_CN), water (H_2_O), glacial acetic acid (CH_3_COOH), regenerated cellulose (RC), triple quadrupole (TQ), electrospray ionization (ESI), selected reaction monitoring (SRM), limit of detection (LOD), limit of quantification (LOQ), apparent recovery (R_A_), signal suppression/enhancement (SSE), recovery of the extraction (R_E_).
